# Seasonal and age-related changes in sperm quality of farmed arctic charr (*Salvelinus alpinus*)

**DOI:** 10.1186/s12864-023-09614-9

**Published:** 2023-09-04

**Authors:** Khrystyna Kurta, Henrik Jeuthe, Rakan Naboulsi, Dirk-Jan de Koning, Christos Palaiokostas

**Affiliations:** 1 Department of Animal Breeding and Genetics, Swedish University of Agricultural Sciences, Box 7090, Uppsala, 750 07 Sweden; 2 Department of Medical Biochemistry and Microbiology, Genetics and genomics, Uppsala University, Uppsala, Sweden; 3 Aquaculture Center North, Åvägen 17, Kälarne, 844 61 Sweden; 4 Department of Women’s and Children’s Health, Karolinska Institute, Tomtebodavägen 18A, Stockholm, 17177 Sweden

**Keywords:** Arctic charr, Sperm concentration, Motility, Season, Age, Whole-genome sequencing

## Abstract

**Background:**

Substantial variation in male fertility is regularly observed in farmed Arctic charr. However, detailed investigations of its fluctuation during a reproductive season and across years are lacking. Furthermore, information about the effect of underlying genetic factors influencing sperm quality is scarce. The current study focused on seasonal and age-related factors that may affect sperm quality characteristics in males reared in natural and delayed photoperiods. Animals were sampled three times for two consecutive years, and sperm quality parameters were recorded using a computer-assisted sperm analysis (CASA) system. Thereafter, high-throughput sequencing technologies were applied, aiming to identify genomic regions related to the variation of sperm quality throughout the reproductive season.

**Results:**

An across-season variation in the recorded sperm quality parameters was evident. Overall, 29% and 42% of males from the natural and delayed spawning groups had a highly variable total progressive motility. Males at four years of age showed significantly higher sperm motility and velocities during the early October and November recordings compared to the following year when the same animals were five years of age. On the other hand, the opposite was observed regarding sperm concentration during the last sampling. A genome-wide *F*_*ST*_ scan detected SNP differentiation among males with high and low variability in total progressive motility (PM) on eight chromosomes (*F*_*ST*_ > 0.17), Genome wide windows with the highest *F*_*ST*_ contained SNPs in proximity (within 250 kb up- and downstream distance) to 16 genes with sperm quality biological functions in mammalian species.

**Conclusion:**

Our findings provide a detailed view of seasonal, age-related, and genetic effects on sperm quality and can be used to guide decisions on broodstock selection and hatchery management.

**Supplementary Information:**

The online version contains supplementary material available at 10.1186/s12864-023-09614-9.

## Background

Arctic charr (*Salvelinus alpinus*) is a cold-water salmonid farmed and selectively bred in Sweden since the 1980s. The main production of Arctic charr is concentrated in Iceland, Sweden, Norway, Canada, and Austria [1]. Currently, Arctic charr farming in Scandinavia is challenged by a high variation in fertilization and hatching rates [2, 3]. These factors limit the industry expansion and predictability of fertilised egg production; and, therefore, require close attention.

Generally, the fertilisation success in fish is greatly affected by the rearing environment [4]. However, high variability in gamete quality and fertilisation rates can be observed within fish groups reared under identical environmental conditions [5, 6]. Prior studies have focused on the effect of egg quality on fertilisation success, highlighting the between family variations in Arctic charr (fertilization: 59–100%, hatching rates: 9–98% [2]). Moreover, a number of research studies have identified a strong relationship between sperm characteristics like concentration and motility and fertilisation in teleosts, supporting their relevance as bioindicators of male fertility [2, 7–11].

Notably, sperm concentration and motility characteristics vary considerably among farmed Arctic charr males. For instance, sperm concentration among sires varied between 2.9 and 9.3 × 10^9^ cells/mL [2], while sperm motility varied between 5 and 99% [12]. Individual differences in sperm quality parameters can be influenced by e.g., animal age [13, 14], sampling time [15–17], social interactions among males [18], animal handling [4], physiological status [7], seminal plasma composition [19], ovarian fluid effects [20, 21] and genetic factors [12].

A decrease in sperm quality with age has been documented in several salmonids such as rainbow trout *Oncorhynchus mykiss* [13], sockeye salmon *Oncorhynchus nerka* [22], and Atlantic salmon *Salmo salar* [23]. Moreover, varying trends in terms of sperm quality have been observed during the reproductive season in farmed fish. More specifically, positive trends in sperm quality across the reproductive season have been observed in rainbow trout [24], red porgy [15], and common carp [25]. In northern pike *Esox Lucius*, sperm quality has been shown to be higher at the beginning and the end of the season than in the mid-season [26]. On the other hand, negative trends have been documented e.g., Atlantic halibut *Hippoglossus hippoglossus* [27]. Furthermore, in Atlantic cod, some studies have shown sperm quality to peak during mid-season and decrease towards the end of the spawning period [28], while in other studies, no apparent change was detected [16] regardless whether the fish were of farmed or wild-caught origin [16, 29]. Notably, potential changes in sperm quality across the same spawning season or due to age have not been documented in Arctic charr. Therefore, understanding the seasonal dynamics of sperm quality, as well as its potential to be manipulated, is crucial to maximizing the reproductive output of a broodstock enabling a resource-efficient and predictable hatchery production.

The genetic architecture of sperm quality has been widely studied in livestock, highlighting a complex polygenic nature [30–32]. As such, the existence of a large number of genes that can affect male fertility has been suggested in bulls [33], stallions [34], and boars [35]. Notably, our recent study on selectively bred Arctic charr [12] revealed a significant genetic component affecting sperm quality characteristics, suggesting that those traits are moderately heritable (0.21–0.32).

The rapid progress in next-generation sequencing technologies has developed powerful platforms allowing for whole genome sequencing (WGS) and detection of millions of genetic variants. At present, WGS has been successfully applied in finding single nucleotide polymorphisms (SNPs) associated with fertility in bulls [36] and can be a valuable tool for discovering genomic regions for fertility in farmed fish. Moreover, using approximately 5,000 SNPs, a genomic region in proximity to a gene with known biological functions related to sperm quality in mammals has been found in Arctic charr [12]. However, the relatively low SNP genotyping density did not cover a large part of the genome. Thus, many genomic regions with a potential effect on sperm quality most likely remained uncovered.

In the present work, we studied the variation of sperm quality across age and within the spawning season in farmed Arctic charr reared under different photoperiods. The following sperm quality bioindicators were assessed: sperm concentration, progressive sperm motility, and sperm kinematic parameters. Both individual and family-based performance in terms of sperm quality was evaluated through their genomic breeding values. Finally, WGS and double digest restriction-site associated DNA sequencing (ddRAD-seq) were applied on males with consistently high (n = 8 WGS; n = 63 ddRAD-seq) versus variable sperm quality (n = 8 WGS; n = 31 ddRAD-seq) across the reproductive season.

## Materials and methods

### Background information of sampled males

Arctic charr males from Aquaculture Centre North (ACN) were used in this study. The station is the core facility for the Swedish Arctic charr breeding program and is located in Kälarne, central Sweden. The breeding program has been operating within a closed breeding nucleus since its start in the 1980s [37]. Pedigree information is recorded using PIT-tags (Passive Integrated Transponder).

**Fig. 1:**
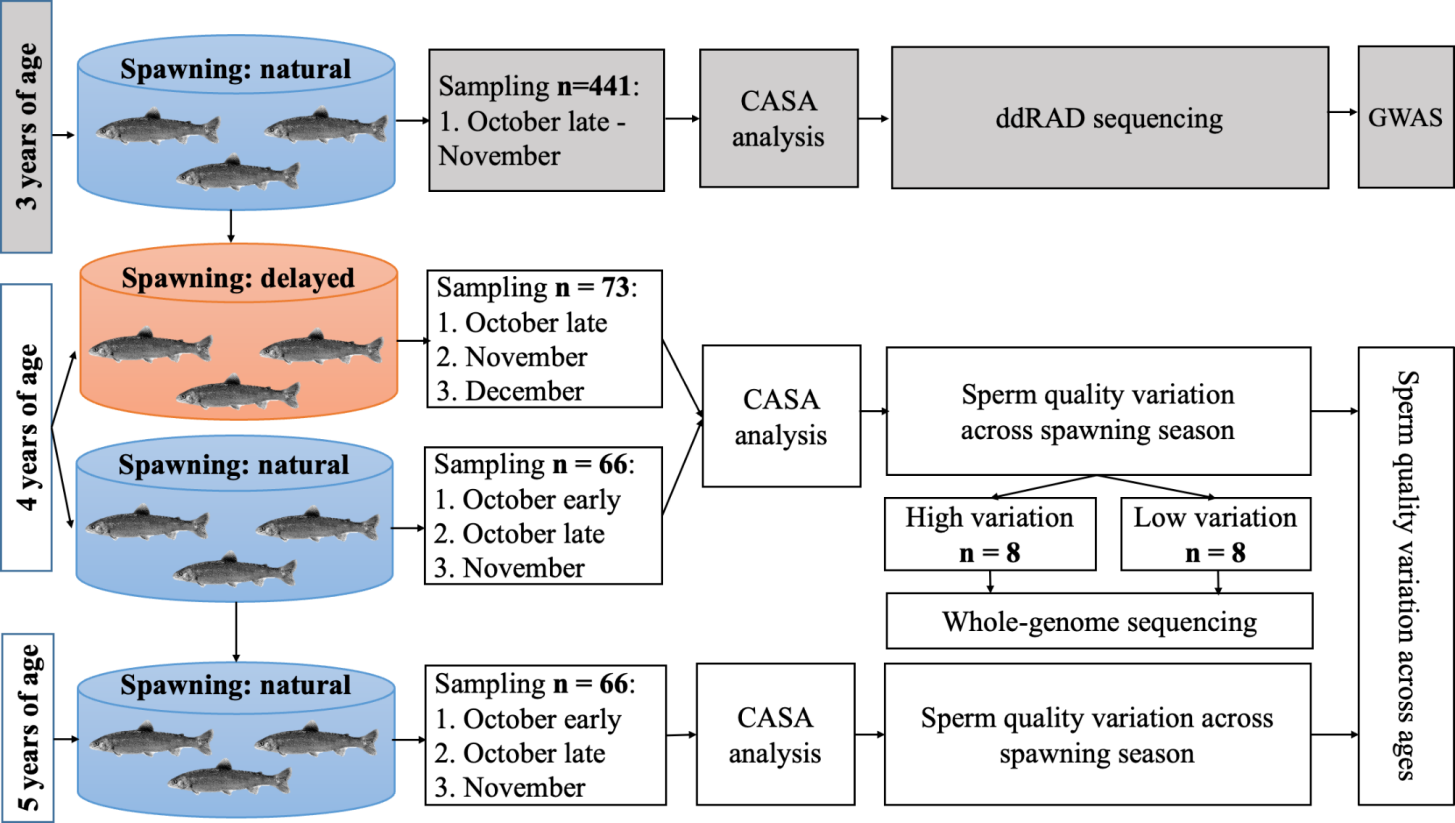
Schematic illustration of the sperm quality assessment in selectively bred Arctic charr (*Salvelinus alpinus*) throughout the spawning season in two consecutive years (2021, 2022). The grey colour indicates the previously published data [12] that were used in the present study

The studied animals originated from the 8th generation of the breeding program and were sampled for two consecutive years when they were at the age of four and five years (the family structure of the sampled animals is represented in Table S1). Moreover, recordings from the same animals at three years of age were a priori available from our previous study [12]. The schematic illustration of the study design is shown in Fig. 1.

All fish were reared in indoor concrete tanks with a flow-through water supply and ambient water temperature. Animals were fed 2% of their body weight daily with Vitalis feed (Skretting, Stavanger, Norway) using an automatic feeding system (Arvo-Tec Oy, Huutokoski, Finland). The sampled animals originated from two groups of natural and delayed spawning, respectively. Specifically, in the latter case, spawning was controlled by manipulating the photoperiod with artificial lights using broad-spectrum fluorescent tubes. Animals in the tanks with delayed spawning had winter extended by 6 weeks with 6 h light, a delayed spring and summer with a natural day length (20 h light), followed by an abrupt shortening of a day length to 6 h in early October. The schedule was based on a previous study on Arctic charr [38] and resulted, as planned, in the postponement of the spawning season by one month, starting at the turn of October-November.

### Sampling

**Fig. 2:**
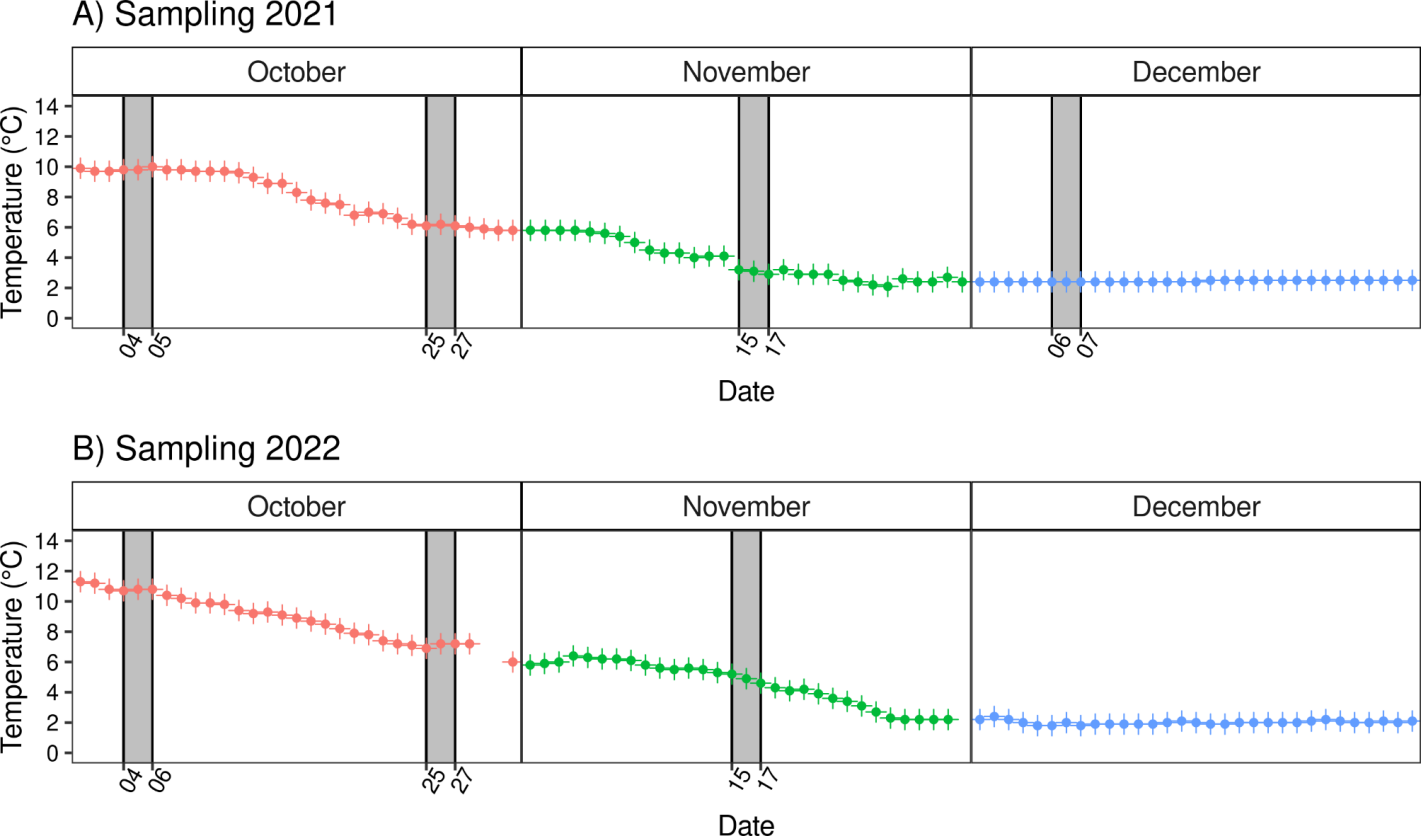
Water temperature variation across the spawning season of farmed Arctic charr (*Salvelinus alpinus*) at sampling in 2021 (**A**) and 2022 (**B**). Grey rectangles indicate the date when the sperm samples were collected from the studied males

Milt sampling was performed repeatedly from Arctic charr males in 2021 and 2022 when the animals were four and five years old, respectively. In particular, when the fish were four years old, n = 66 males were sampled from the naturally spawning group and n = 73 males from the delayed group. In the subsequent season, when these males were five years of age, n = 66 males were sampled from the natural spawning group. To track the variability in sperm quality, milt was collected from the same individuals three times across the spawning season. More precisely, the season was divided into three parts: early October (4th – 5th), late October (25th – 26th), and November (15th − 16th) for animals with natural spawning; and late October (27th), November (16th − 17th) and December (6th – 7th) for animals with delayed spawning (Fig. 2). The followed sampling schedule reflects the period when the majority of artificial stripping is performed at the facility.

The water temperature during the sampling period varied between 2.4 and 11.3 °C (Fig. 2). Prior to sample collection, fish were anesthetized using *MS-222* (Sigma Aldrich, St. Louis, MO, USA). On each sampling occasion, milt samples were collected through manual stripping in individual disposable cups over a maximum of one hour from the first to the last male and stored at 4 °C.

### Recordings of sperm concentration, motility, and velocity

Measurements of sperm concentration, motility, and velocity were performed on the same day at the on-site laboratory of ACN. A computer-assisted sperm analysis (CASA) system equipped with the SCA® Motility imaging software (Microptic, Spain) was used for recording sperm motility and kinematic parameters. The installation and threshold parameter adjustment suitable for analysing salmonid sperm, including Arctic charr, were performed by the manufacturer. The minimum velocity for motile sperm was set to VCL ≥ 20 μm/s. The CASA system setup, composition, and detailed procedure were previously described by Kurta et al. [12] and presented in supplementary Table S2. In short, CASA measurements for each sample were taken 2–3 times using standardised 20 μm-depth slides with two counting chambers (CellVision, Heerhugowaard, Netherlands). Before loading a sample, the slides were pre-cooled at 8°C. Sperm motility and velocity were assessed at 15 s following activation with ActiFish (IMV Technologies, L’Aigle, France) using a ratio 1:10. The image capture configurations used for the analysis were set to record every 5 s at a frame rate of 100 fps (50 frames) with one field per sample being analyzed. As sperm in salmonids tends to remain active for approximately 30s recoding multiple fields from the same sample was not possible. Overall, recordings were based on > 100 cells for each animal. The following CASA parameters were recorded: progressive sperm motility (PM, %), average path velocity (VAP, µm/s), curvilinear velocity (VCL, µm/s), and straight-line velocity (VSL, µm/s).

Sperm concertation (SC, ×10^9^ cells/mL) was measured using the NucleoCounter® SP-100™ (Chemometec, Denmark). Briefly: 20 µL of sperm sample were diluted with 20 mL lysis buffer Reagent S-100 (Chemometec, Denmark), with the measurement performed using settings for bull semen according to the manufacturer’s recommendations [2, 39].

In addition, the present study included the sperm quality recordings from 2020 measured on the same animals and following the same procedure [12] as described above. However, sperm motility parameters were recorded using water as an activation solution instead of ActiFish. Therefore, no direct comparisons between sperm motility recordings taken in 2020 with motility-related traits from 2021 or 2022 were possible.

### Descriptive statistical analysis

Descriptive statistics were computed for the sperm quality parameters using R v.4.0.2 [40]; the R/packages *tidyr* and *dplyr* [41], and *ggpolt2* [42]. Variations of sperm quality across the reproductive season were analyzed by non-parametric tests for comparing either two (Wilcoxon test) or multiple groups (Kruskal-Wallis test). In addition, linear mixed effects models were fitted to estimate the significance of sampling time and photoperiod (model 1). Furthermore in model 2 the interaction between sampling time and photoperiod was also included, while in model 3, age was included as well (supplementary Table S4, Table S5). Models were fitted via the lme4 [43] and lmerTest (to estimate *P* values; [44]) packages in R statistical software [40]. Model 1 and model 2 were fitted for records of males from 2021 spawning year with natural and delayed spawning. Model 3 was fitted for records of males from 2021 to 2022 spawning years with natural spawning. Finally, Pearson or Spearman correlations were used to determine relationships between the sperm quality parameters. Pearson coefficients were used for sperm concentration and velocities, while Spearman rank correlation was used for the total progressive motility.

### Selection of animals for whole-genome sequencing analysis

Total progressive motility (PM) was chosen to assess individual variability during the spawning period. This parameter is valuable for objective sperm assessment as it highly correlates (*r* ≈ 0.95, *P* < 0.001) with other sperm quality traits (e.g., VCL, VSL, VAP) and fertilisation rates (r ≈ 0.6–0.9; Gallego and Asturiano 2018b). A suggestive threshold for PM of > 80% was considered as indicator of high-quality sperm [7]. As such, samples were divided into two groups: (i) animals with low variability (CV < 0.3) and across season mean PM > 80%; and (ii) animals with high variability (CV > 0.36) and across season mean PM < 70%. In total, 16 males were selected for the whole-genome sequencing: samples with the high (n = 8) and the low sperm quality (n = 8) in terms of PM from both the natural and delayed spawning groups.

### DNA extraction and whole-genome sequencing

Genomic DNA was extracted from 16 sperm samples using a salt-based precipitation method following the protocol described by Palaiokostas et al. [45]. In short, fin tissue was digested at 55 °C for 4 h using a lysis solution containing 200 µL SSTNE (50 mM Tris base, 300 mM NaCl, 0.2 mM each of EGTA and EDTA, 0.15 mM of spermine tetrahydrochloride, and 0.28 mM of spermidine trihydrochloride; pH 9; Sigma-Aldrich, Darmstadt, Germany), containing 10% SDS (Bio-Rad, Hercules, USA), and 100 µg proteinase K. After the digestion, 5 µL RNaseA (2 mg/mL; Thermo Fisher, Vilnius, Lithuania) was added, and the samples were incubated at 37 °C for 60 min. Proteins were precipitated by adding 0.7 volume of 5 M NaCl (Sigma-Aldrich, Darmstadt, Germany). The genomic DNA was pelleted by the addition of 0.7 volume of isopropanol and centrifugation (Pico 21, Thermo Fisher, Waltham, MA, USA) at 14,000 g for 5 min. Following overnight incubation with 75% ethanol, the DNA pellet was dissolved in 30 µL of 5 mM Tris (pH 8.0; Sigma-Aldrich, Darmstadt, Germany). The purity of the extracted DNA was assessed by spectrometry using a NanoDrop 8000 (Thermo Fisher, Waltham, USA). DNA concentration was measured on Qubit 2.0 fluorometer using the dsDNA Broad Range Assay Kit (Invitrogen, Life Technologies, Grand Island, USA).

Extracted DNA samples were sent to the National Genomics Infrastructure center in Uppsala, Sweden. The quantity and integrity of the extracted DNA were assessed at the center using a TapeStation system in conjunction with the Genomic DNA ScreenTape assay (Agilent Technologies, Waldbronn, Germany). The TruSeq DNA PCR-free kit (Illumina, San Diego, USA) was used for library preparation, followed by whole-genome sequencing in an Illumina NovaSeq6000 instrument using one lane of an S4 v1.5 flow cell with a read setup of 2 × 150 cycles.

### Whole-genome sequencing data processing, variant calling, and filtering

The raw reads (Q > 30) were mapped to the Salvelinus sp. reference genome assembly (GenBank accession number GCF_002910315) using the bwa-mem algorithm [46]. Duplicated reads were removed with Picard MarkDuplicates [47]. The average sequencing coverage depth was 19X (range: 17-29X). Quality control of the mapped reads and variant calling were performed following the Genome Analysis Toolkit (GATK) best practice recommendations. In particular, HaplotypeCaller and GenotypeGVCFs from GATK v4.1.4.1 [48] were used to identify biallelic SNP genotypes. Thereafter, hard filtering was applied according to GATK recommendations using seven parameters: QualByDepth (QD), FisherStrand (FS), RMSMappingQuality (MQ), MappingQualityRankSumTest (MQRankSum), ReadPosRankSumTest (ReadPosRankSum). Following the GATK guidelines, SNPs that did not meet the specified criteria: QD > 2.0, QUAL > 30.0, SOR > 5.0, FS < 60.0, MQ > 40.0, MQRankSum>–12.5, ReadPosRankSum>–8.0, were removed by GATK VariantFiltration tool. The retained variants were filtered for minor allele frequency (MAF) above 0.05 and a calling rate of 100% with VCFtools v0.1.16 [49]. After the filtering, 6,774,790 SNPs were retained for the downstream analysis.

### ddRAD genotypes

Genotypic information (5418 SNPs) identified with ddRAD-seq was available for n = 103 males from our previous study [12]. In addition, genomic estimated breeding values were previously obtained for all sampled males based on sperm quality records taken between late October and November 2020 [12].

### Fixation index (*F*_*ST*_) estimates and analysis of gene ontology

To evaluate the degree of genetic differentiation between the two cohorts, VCFtools functions --fst-window-size and --fst-window-step were used to calculate the genome-wide *F*_*ST*_ in 200 kb windows with a 100 kb window step along the genome. The average SNPs density per window was 563 ± 280 SD. In the subsequent gene ontology analysis, SNPs from the windows with the highest *F*_*ST*_ were tested for proximity to genes with functions relevant to sperm quality. More specifically, the existence of candidate genes was searched within the 250 kb up- and downstream regions of these SNPs using the NCBI gene annotation file for the *Salvelinus sp.* [Genbank accession number GCF_002910315.2]. Information on the gene function of the detected genes was obtained from the literature. Furthermore, *F*_*ST*_ estimates were obtained from the a priori available ddRAD data containing information from 5,418 SNPs [12]. In this case *F*_*ST*_, based on single SNPs, was calculated using the VCFtools function --weir-fst-pop. Finally, the 99.5% quantile cutoffs of the *F*_*ST*_ empirical distributions from the WGS and the ddRAD data were used as a naive estimate of significance.

## Results

### Variation of sperm quality within reproductive seasons

**Fig. 3:**
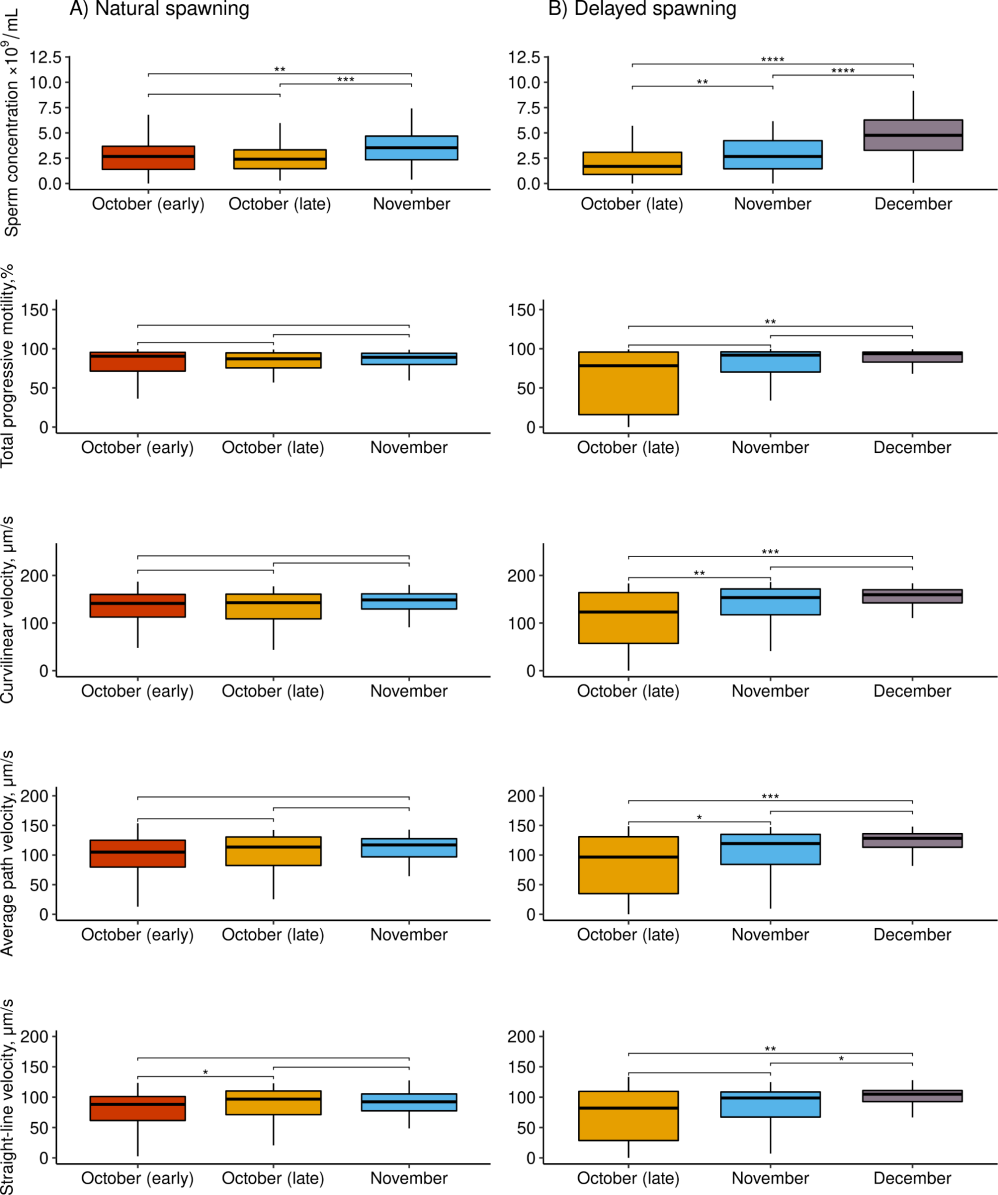
Sperm quality parameters of Arctic charr (*Salvelinus alpinus*) males at different sampling points across the 2021 spawning period in groups of natural (**A**) and delayed (**B**) spawning. Brackets with asterisks indicate statistically significant differences (Wilcox test, *P < 0.05, **P < 0.01, ***P < 0.001)

Descriptive statistics for the sperm quality parameters measured in the natural and delayed spawning groups are summarised in Fig. 3 and supplementary Table S3. A positive trend over time was observed regarding the mean sperm quality traits in both groups. More specifically, in the natural spawning group, sperm concentration increased by more than 30% between the first and last sampling points in both years. Furthermore, sperm motility parameters increased by 10–54% across sampling points (Table S3). However, the differences in motility-related traits between sampling occasions were significant only for the 2022 measurements.

In the delayed group, sperm concentration increased by 39% (*P* < 0.001) from October to November and 133% (*P* < 0.001) from October to December. At the same time, an increase of 14–45% was observed in motility traits from October to December (significant for VAP, VSL, *P* < 0.05; Fig. 3).

**Fig. 4:**
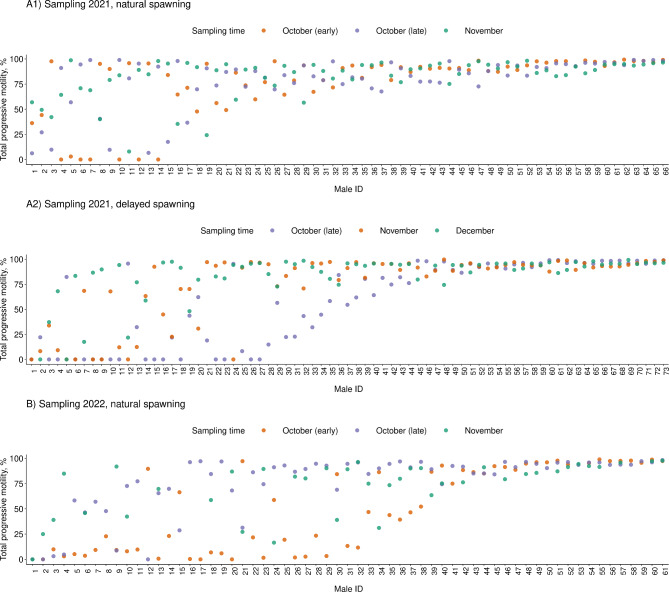
Total progressive motility in farmed Arctic charr (*Salvelinus alpinus*) males at different times (indicated by point colour) across the spawning periods in 2021 (**A**) and 2022 (**B**) in the groups with natural and delayed spawning

In terms of the 2021 sampling, 65% (n = 43) of the studied males from the natural spawning group had a high PM with relatively low variability across sampling points (mean PM: 80–98%; SD: 0–21%; CV: 0.01–0.26); while 29% of the males (n = 19) had a low and highly variable PM (mean PM: 33–70%; SD: 12–55%; CV: 0.11–0.91) across the three sampling points. At the same time, 37% (n = 7) of these males showed an increase in terms of PM toward the end of the sampling time, while the opposite pattern was observed for 32% (n = 6) of those. Moreover, regarding the delayed spawning group, about 51% of males (n = 37) exhibited limited variability in PM across the monitoring period (mean PM: 81–98%; SD: 1–23%; CV: 0.01–0.28), while 42% (n = 31) had relatively high variability in PM (mean PM: 0–70%; SD: 11–65%; CV: 0.13–1.78). Among the males with highly variable PM, 58% (n = 18) showed an increase, and 10% (n = 3) showed a decrease in PM across the sampling points (Table S6, Fig. 4).

During the 2022 sampling, 39% (n = 24) of the studied males from the natural spawning group had a high PM with relatively low variability (mean PM: 80–98%; SD: 0–24%; CV: 0-0.30), while 56% (n = 34) had a low and highly variable PM (mean PM: 0–69%; SD: 14–69%; CV: 0.36–1.42) across the three sampling points. In the latter group, 37% (n = 24) of males showed an increase in PM toward the end of the sampling period, while fewer males (5%, n = 3) showed the opposite pattern (Table S6, Fig. 4).

Notably, 64% (*n* = 29) of the families that had records from at least two males (n = 45) in both the natural and the delayed spawning groups exhibited relatively similar performance in terms of the PM (mean PM difference below 30%) across the spawning season. The remaining 36% (n = 16) of the families included males that showed considerable (above 30%) differences in mean PM during the sampling period.

Additionally, moderate to high correlations (0.31–0.67, *P* < 0.01) were found for sperm concentration measured across both spawning seasons in the case of the natural spawning group (Table 1). In the case of motility-related parameters, lower correlation coefficients were obtained during the 2021 reproductive season in the natural spawning group compared to the ones from the subsequent year. For the former, correlations ranging between − 0.01 and 0.26 were estimated. On the other hand, moderate to high (0.25–0.67) correlations were found during the 2022 reproductive season when the same animals were five years of age (Table 1).

**Table 1:** Correlation coefficients between sperm quality parameters measured across the spawning season in Arctic charr (*Salvelinus alpinus*) males with natural and delayed spawning

Trait	Natural			Delayed		
	October early vs. October late	October early vs. November	October late vs. November	October late vs. November	October late vs. December	November vs. December
**2021**						
SC	0.57^***^	0.53^***^	0.68^***^	0.66^***^	0.31^**^	0.67^***^
PM	-0.01	0.04	0.19	0.43^***^	0.22	0.50^***^
VCL	0.04	0.16	0.26^*^	0.46^***^	0.19	0.54^***^
VAP	0.09	0.17	0.23	0.42^***^	0.19	0.51^***^
VSL	0.08	0.12	0.18	0.41^***^	0.19	0.51^***^
**2022**						
SC	0.42^**^	0.39	0.47^***^	-	-	-
PM	0.43^***^	0.25	0.55^***^	-	-	-
VCL	0.39^**^	0.34^**^	0.67^***^	-	-	-
VAP	0.41^***^	0.32^*^	0.66^***^	-	-	-
VSL	0.38^**^	0.29	0.67^***^	-	-	-

SC: sperm concentration, ×10^9^ cells/mL; PM: total progressive motility, %; VCL: curvilinear velocity, µm/s; VAP: average path velocity, µm/s; VSL: straight-line velocity, µm/s. *P < 0.05, **P < 0.01, ***P < 0.001

The fitted mixed effect model showed that sampling time and photoperiod had a statistically significant effect (P < 0.05) on sperm quality characteristics (Table S4). Moreover, the parameter signs were positive when a model without interaction between sampling time and photoperiod was fitted. Finally, when a model including the interaction between sampling time and photoperiod was fitted, the latter had a significant effect (*P* < 0.01) for sperm concentration, while for motility-related traits the estimates were significant (*P* < 0.001) for the delayed group and sampling in late October.

### Variation of sperm quality between reproductive seasons

**Fig. 5:**
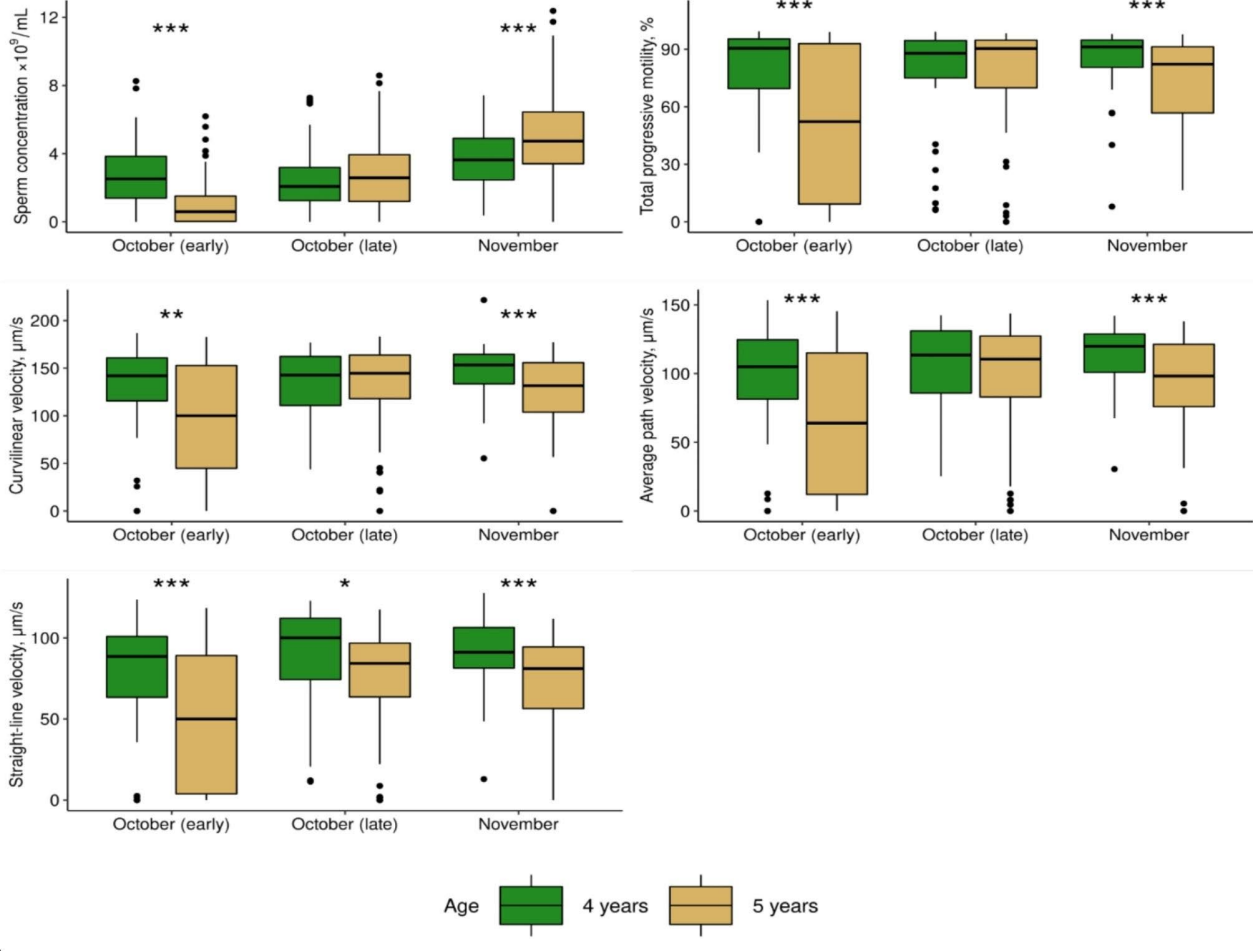
Sperm quality parameters in farmed Arctic charr (*Salvelinus alpinus*) males at the age of four and five years from the group of the natural spawning. Asterisks indicate means for the sperm quality parameters that were significantly different (*t*-test, *P < 0.05, **P < 0.01, ***P < 0.001)

Sperm concentration on average was higher in males of four years of age at the beginning of the spawning season, while in the mid- and late season, higher sperm concentrations were obtained when the animals were five years old. Furthermore, sperm motility parameters were on average higher when the animals were four years of age compared to the recordings on the same animals in the following spawning season. In addition, males at the age of five showed higher variability in progressive motility and velocity at the beginning of the reproductive season (SD: 39–58) compared to the previous spawning season (SD: 30–47). Overall, the differences between sperm quality parameters of the same animals between the two reproductive seasons were statistically significant (*P* < 0.05) at the beginning (early October) and at the end of the season (November) (Table 1; Fig. 5).

Moreover, Pearson correlations between 0.21 and 0.69 were estimated for sperm quality parameters between the two reproductive seasons in the case of the natural spawning group (Table 2). More specifically, sperm concentrations showed high and significant (*P* < 0.01) correlations in both late October and November recordings. Regarding the motility-related traits, moderate and statistically significant correlations were found during the early (*P* < 0.05) and late October (*P* < 0.01) recordings. In the case of the last recording, a statistically significant correlation was found only for VSL (*P* < 0.05).

**Table 2:** Correlations between sperm quality parameters measured across four and five years of age in farmed Arctic charr (*Salvelinus alpinus*) males from the natural spawning group

Parameter	October early	October late	November
SC	0.24	0.54^***^	0.69^***^
PM	0.27^*^	0.32^***^	0.21
VCL	0.31^*^	0.37^***^	0.28
VAP	0.32^**^	0.38^***^	0.29
VSL	0.29^*^	0.36^***^	0.33^*^

SC: sperm concentration, ×10^9^ cells/mL; PM: total progressive motility, %; VCL: curvilinear velocity, µm/s; VAP: average path velocity, µm/s; VSL: straight-line velocity, µm/s. *P < 0.05, **P < 0.01, ***P < 0.001

Fitting a mixed effect model including the age parameter on the examined sperm quality characteristics was deemed statistically significant (*P* < 0.001) for all motility-related traits; however no significant effect was found for sperm concentration. Finally, the obtained parameters were negative for motility and velocities (Table S4).

### Associations between sperm quality traits and GEBVs

Available GEBVs from our previous study [12] were used to assess the relationships between predicted breeding values based on sperm quality recordings when the animals were three years of age and the sperm quality recordings of the current study.

In the case of the 2021 reproductive season, the genomic estimated breeding values exhibited moderate (*r* ~ 0.4, *P* < 0.01) correlations with observed sperm concentration across the spawning season, with the exception of the early October sampling point on the natural spawning group (*r* ~ 0.2, *P* > 0.05). In terms of total progressive motility, correlations were low and not significant (r = 0.13 − 0.19, *P* > 0.05). Additionally, observed velocities showed low to moderate correlations (*r* = 0.15–0.34) with the GEBVs. Significant associations were found only during the last sampling point in the natural spawning group (*r* ~ 0.3, *P* < 0.05). On the other hand, in the case of the delayed spawning group, the estimated correlations between the GEBVs and PM or velocities ranged between − 0.09 and 0.20 with *P* > 0.05 (Table 3).

As of the 2022 reproductive season, moderate to high correlations (*r* ~ 0.48–0.67, *P* < 0.05) were observed between GEBVs and sperm concentration in the first two sampling points, while a low and non-significant correlation was found for the last measurement. As in the case of the 4-year-old males, significant and moderate correlations between GEBVs and all velocity parameters were found in November (*r* ~ 0.28–0.47, *P* < 0.05), while no significant correlations were found for total progressive motility. On the other hand, all correlations were moderate and negative between GEBVs and the motility or velocity traits that were measured in early October (*r* ~ − 0.28 to − 0.39, *P* > 0.05).

**Table 3:** Correlation coefficients between sperm quality parameters and genomic estimated breeding values in farmed Arctic charr (*Salvelinus alpinus*) males across spawning seasons

Parameter	Natural spawning			Delayed spawning		
	October early	October late	November	October late	November	December
**2021**						
SC	0.20	0.45^***^	0.43^***^	0.41^**^	0.41^**^	0.40^**^
PM	0.13	0.19	0.13	-0.04	0.09	0.20
VCL	0.15	0.25	0.30^*^	-0.07	0.20	0.17
VAP	0.23	0.25	0.34^*^	-0.09	0.19	0.17
VSL	0.20	0.20	0.29^*^	-0.02	0.20	0.19
**2022**						
SC	0.48^*^	0.67^***^	0.09	-	-	-
PM	-0.30	0.12	0.36	-	-	-
VCL	-0.28	0.19	0.28^*^	-	-	-
VAP	-0.35	0.25	0.45^**^	-	-	-
VSL	-0.39	0.30	0.47^**^	-	-	-

SC: sperm concentration, ×10^9^ cells/mL; PM: total progressive motility, %; VCL: curvilinear velocity, µm/s; VAP: average path velocity, µm/s; VSL: straight-line velocity, µm/s. *P < 0.05, **P < 0.01, ***P < 0.001

### Whole-genome sequencing and genome-wide *F*_*ST*_

The genome-wide fixation coefficient (*F*_*ST*_) was calculated over a sliding window of 200 kb with 100 kb steps between males of high and low variability in total progressive sperm motility across the reproductive season. Animals from the highly variable group had a mean PM ranging between 35 − 70% and a standard deviation of SD = 40 − 56. The low variable group comprised animals with a mean PM between 95 − 98% and SD = 1 − 3.

**Fig. 6:**
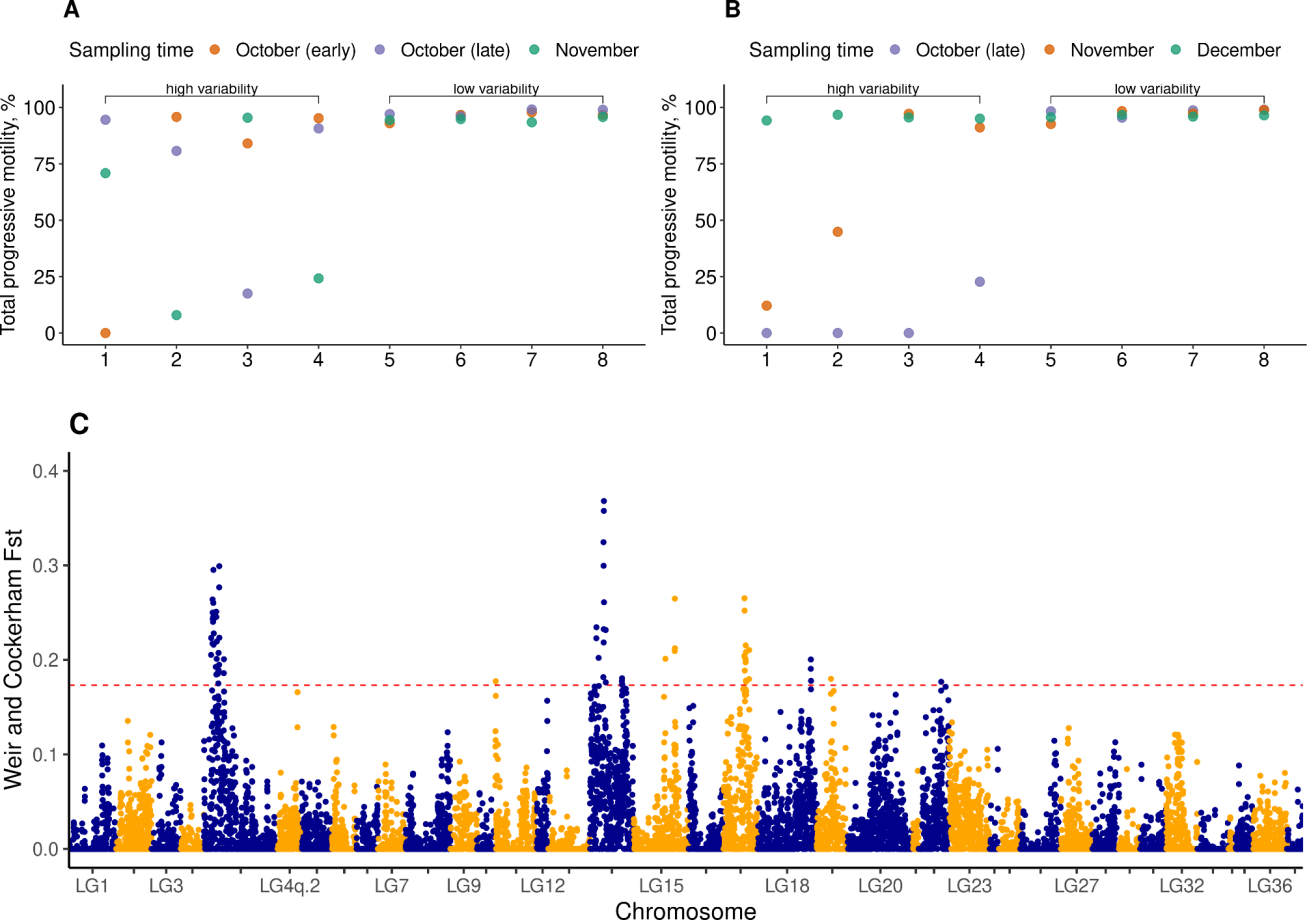
Manhattan plot showing the Weir and Cockerham’s *F*_*ST*_ values in 200-kb windows along the whole genome (plot C) between Arctic charr (*Salvelinus alpinus*) males with high (*n* = 8) and low (*n* = 8) variability in total progressive motility in the natural (**plot A**) and delayed (**plot B**) spawning groups. The x-axis indicates the chromosome where the SNPs were located, and the y-axis denotes the *F*_*ST*_ value. The red solid line represents the 99.5% percentile cutoff

The 99.5% percentile cutoff of the *F*_*ST*_ empirical distribution was 0.17. *F*_*ST*_ values exceeding the cutoff were observed on eight chromosomes: LG4q.1:29, LG11, LG14, LG15, LG17, LG18, LG19, LG22 with the highest values on LG14 (*F*_*ST*_ = 0.37, Fig. 6).

SNPs on windows with the highest *F*_*ST*_ on each chromosome were tested for proximity to genes (250 kb upstream and downstream distance). Overall, 36 genes were found across the four chromosomes (three on LG4q.1:29, nine on LG11, 13 on LG14, and 11 on LG22). Among them, 17 genes were previously related to sperm quality in mammalian species, and 19 genes had unknown biological functions (Table 4).

**Table 4:** Genomic windows with the highest Weir and Cockerham *F*_*ST*_ values, SNPs count per window, and genes within 250 kb up- or downstream genomic regions detected in Arctic charr (*Salvelinus alpinus*) male groups with the high and low variability in total progressive motility across the spawning season

Chromosome	SNP count	*F*_*ST*_	Genomic window, bp	Genes
LG4q.1:29	737	0.29	18,800,001–19,000,000	***ASTN2*** [62], ***ZNF618*** [57], ***PAPPA*** [50]
LG4q.1:29	763	0.29	11,600,001–11,800,000	**-**
LG11	500	0.18	500,001–700,000	***BLCAP*** [59], *CHD6, RBM39, CPNE3*, ***FER1L4*** [58], *NFS1*, ***PSMF1*** [53], ***RBM12*** [54], *TMEM74B*
LG14	677	0.37	17,200,001–17,400,000	*PRSS27*, ***FYN*** [51], *CYP39A1*, *RCAN2, SLC22A6, LAMA4*, ***MARCKS*** [63], *PTRHD1*, ***HDAC2*** [55], *MSRA*, ***SLC25A27*** [52], *TUBE1*, ***WISP3*** [56]
LG15	531	0.27	50,300,001–50,500,000	**-**
LG17	361	0.27	25,700,001–25,900,000	-
LG18	418	0.20	65,400,001–65,600,000	-
LG19	1336	0.18	17,400,001–17,600,000	-
LG22	519	0.18	27,600,001–27,800,000	***VASP*** [60], *TRAPPC4, SMD2*, ***DACT1*** [61], ***NBGRP7*** [54], *CLMP*, ***C5AR1*** [65], *BARHL2*, *FOXG1, GRAMD1B*,* CLTC*

Genes with the biological function linked to sperm quality in mammalian species are depicted in bold.

In particular, the protein coding genes that potentially play role in spermatogenesis and sperm function included: *PAPPA* [50], *FYN* [51], *WISP3* [52], *PSMF1* [53], *RBM12* [54], *NBGRP7* [54]; involved in fertility regulation: *HDAC2* [55], *VASP* [56], *ZNF618* [57], *FER1L4* [58]; *BLCAP* [59] may be associated with sperm DNA damage; found to be expressed in spermatozoa: *DACT1* [60] and *C5AR1* [61]; *ASTN2* [62] is involved to calcium ion binding and potentially related to sperm storage duration in chicken; *MARCKS* coding proteins are involved to calcium mobilization during the acrosomal reaction [63], and its proteins are present in testis and mature spermatozoa in rats [64]; *CLTC* can be involved in sperm production [65].

**Fig. 7:**
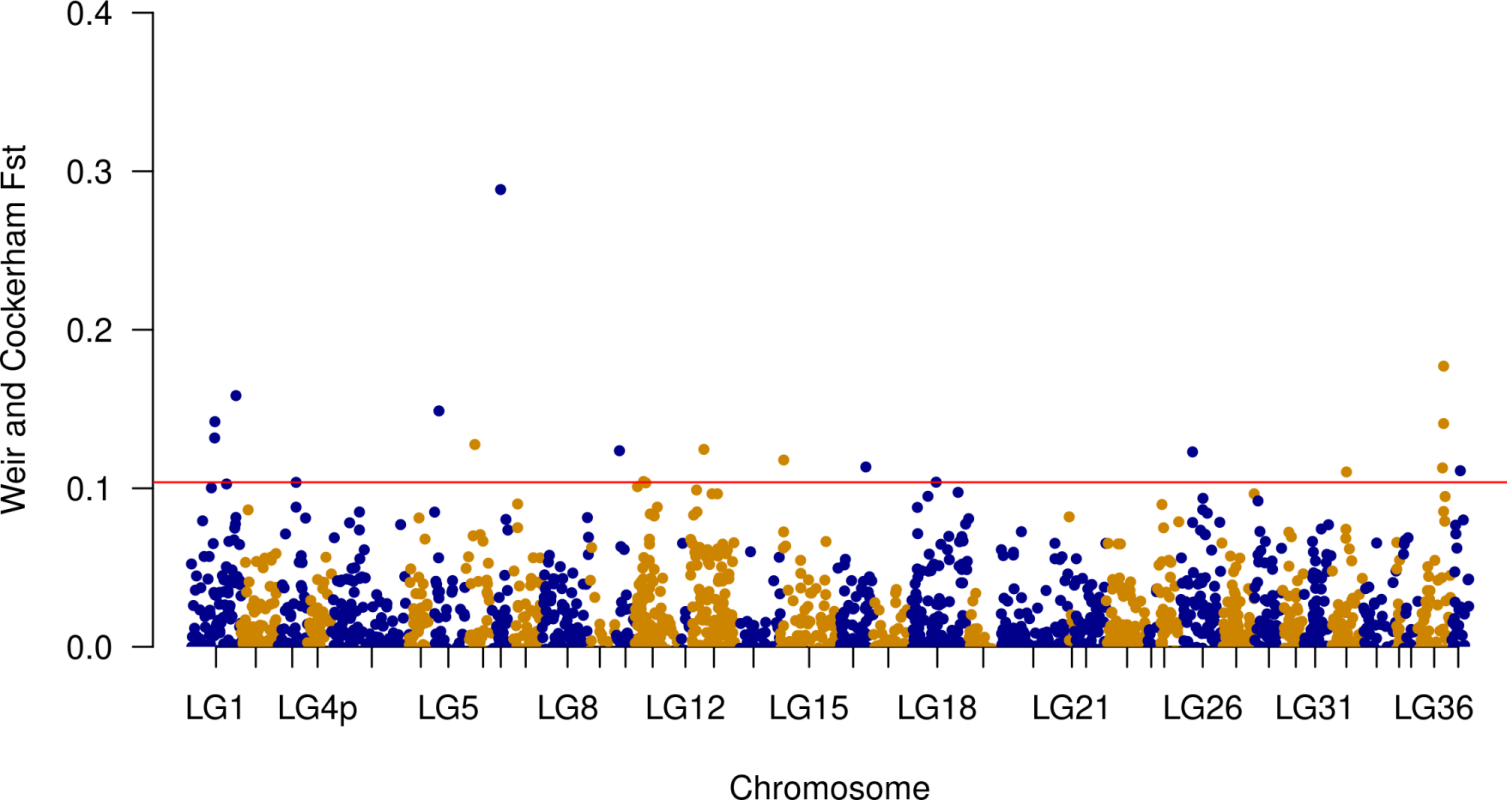
Manhattan plot showing the Weir and Cockerham’s *F*_*ST*_ values of each SNP identified with ddRAD-seq between Arctic charr (*Salvelinus alpinus*) male groups of high (*n* = 31) and low (*n* = 63) variability in total progressive motility. The x-axis indicates the chromosome where the SNPs were located, and the y-axis denotes the *F*_*ST*_ value. The red solid line represents the 99.5% percentile cutoff of the empirical distribution

In addition, the *F*_*ST*_ index based on single SNPs was calculated for 5418 SNPs previously identified by Kurta et al. (2022) using ddRAD-seq. Genotypes were available for 103 samples in total. Out of these males, *n* = 31 males had a relatively high and n = 63 a low seasonal variability in total progressive motility from the natural and delayed spawning groups (Table S2, Fig. 4). *F*_*ST*_ values exceeding the 99.5% quantile cutoff were identified on 12 chromosomes: LG1, LG5, LG6.1, LG6.2, LG10, LG11, LG13, LG15, LG16, LG18, LG26, LG32, LG36, LG37 (Fig. 7). The largest *F*_*ST*_ (0.29) was found on chromosome LG6.2. Overlapping regions between the WGS and ddRAD datasets were observed on chromosomes LG11, LG15, and LG18.

## Discussion

Understanding the genetic basis of sperm quality changes during the breeding period would support the management of Arctic charr hatcheries. In the present work, seasonal and age-related variations in sperm quality parameters during the spawning season in farmed Arctic charr were characterised for the first time on both individual and family levels. The observed variation in sperm quality parameters can explain, to a certain degree, the highly variable reproductive performance of Arctic charr in captivity [2, 12]. The above is more apparent in selective breeding designs where single males are used to fertilise individual egg batches. Moreover, this is the first report on whole-genome sequencing aiming to identify genomic regions associated with changes in Arctic charr sperm quality over the reproductive season.

### Across season variation of sperm quality parameters

It is commonly considered that sperm cells retain high quality in the testes throughout the spawning season, while females have a generally shorter reproduction window [66]. However, the latest studies in different fish species showed that male reproductive patterns across the breeding period vary within and between species [15–17]. More specifically, an overall increase until mid-season is reported, and after reaching its peak a downward trend is observed due to changes in hormonal regulation [67] and sperm aging [16, 27, 68–70]. Along with that, the multivariate mixed-effect model analysis indicated significant effects (*P* < 0.01) for sampling time during the spawning season as well as an effect of photoperiod manipulation and spawning year on sperm quality characteristics.

The across-season variation in the recorded sperm quality parameters was evident, particularly in the case of sperm concentration, showing a positive trend in the latter sampling points in both the natural and delayed spawning groups. Notably, the obtained values for sperm concentration (2.01–4.68 × 10^9^/mL) were within a range of values previously reported for the same Arctic charr strain (2.4–9.3 × 10^9^cells/mL; [2, 12]) and for other salmonids (4.7–16.6 × 10^9^/mL; [7]). Sperm concentration showed a significant increase from the beginning until the end of the sampling time in both groups. A similar trend has been demonstrated in other fish species where sperm concentration increased during the spawning season, as in rainbow trout [24], red porgy [15], Atlantic halibut [27], and landlocked Atlantic salmon *Salmo salar* [71]. The increase in sperm concentration throughout the breeding period can be related to increased plasma levels of luteinizing (LH) or follicle stimulating (FSH) hormones [67]. While FSH has been mainly showed to participate in the mitotic phase of spermatogenesis, LH has been widely reported to induce steroidogenesis that results in spermiation and seminal fluid production [15, 67, 72, 73]. As it was previously shown in Arctic charr [74] and other species like striped bass *Morone saxatilis* [73], LH has low levels during spermatogenesis but reaches its peak during spawning. Though, plasma levels of FSH are generally reported high during spermatogenesis with a drop towards the beginning of spawning [75, 76].

An improvement in sperm motility and velocities was generally observed amongst the sampling points in both spawning groups. At the same time, it was statistically significant only for males of the delayed spawning (Table 1; Fig. 3). In addition, a higher variation of the recorded traits was observed in these males compared to the recordings from the natural spawning one. This may be explained by the fact that spermatozoa acquire the capacity for forward motility and fertilization ability in the sperm duct in response to maturation inducing-hormone 17,20β-dihydroxy-4prehnen-3-one (17,20β-P) production along with changes in pH and ionic composition of seminal fluid [67, 77–79]. In Arctic charr, a higher level of 17,20β-P production toward the end of the spawning season was previously reported [2, 80]; and can be considered as the possible reason for the increase in sperm motility and velocity as the spawning season progresses.

### Individual and family-based performance across the spawning season

A primary focus of our study was to gain insights regarding the possibility that the variation of sperm quality parameters during the reproductive season is partially influenced by genetic factors. Overall, 29% and 42% of the animals from the natural and delayed spawning groups, respectively, had a highly variable total progressive motility (mean PM < 70%, CV: 0.13–1.78) across the sampling periods, as compared to other males. Among these males, 32% (natural spawning) and 10% males (delayed spawning) had a drop in sperm quality at the end of the sampling time. Although the reason for that remains poorly understood, environmental and genetic factors are considered as crucial contributors to male reproductive performance [12, 81]. Since all animals were kept under similar conditions, and temperature during sperm sampling on each separate occasion was similar for all males, the drop in sperm quality and, therefore, male fertility can be attributed most likely to genetic differences. To support this claim, males from 29 families out of 45 families represented by more than two males showed similar performance in terms of total progressive motility regardless of whether they were being reared under natural or delayed spawning conditions. Moreover, in our previous study, it was reported that there is a substantial genetic component affecting sperm quality in Arctic charr, and heritability for sperm quality traits is *h*^*2*^ ~ 0.21–0.32 [12]. As such, the considerable proportion of males found here to have a highly variable sperm quality can have a negative impact on the annual hatchery production and the management of the breeding program, especially in terms of maintaining ample genetic diversity in the breeding nucleus. Taking also into account the narrower time frame within which females usually spawn, the above is even more evident. Given that, further studies investigating the genetic causes of low fertility in Arctic charr males empowered by a larger sample size are needed.

### Changes of sperm quality across age

Age-dependent fertility was assessed by sampling and analyzing sperm from the same Arctic charr breeding males during two consecutive spawning seasons. To our knowledge, the variability in sperm quality across ages has not been previously studied in Arctic charr.

Age-related changes in sperm quality have been found in other salmonid fish, such as rainbow trout [13] and sockeye salmon [22] from wild populations. More specifically, results from rainbow trout suggested that 1-2-year-old males had a higher sperm concentration as compared to 3-year-old ones [13]. The same pattern was also observed in sockeye salmon, where the younger (3-year-old) males had a higher sperm concentration than older (5-year-old) ones [22]. However, the results of the above studies are not fully comparable to ours as they relied on wild-captured animals. In addition, 3-year-old males of striped bass had higher sperm concentration than 1-year-old ones [82]. This trend was consistent with our results when comparing sperm concentrations of the last sampling point (November) Nevertheless, the opposite trend was observed during the first sampling point (October).

In terms of the motility-related traits, a more clear pattern was observed, with animals having consistently higher values during the 2021 reproductive season. Compared to previous studies in rainbow trout [13] and sockeye salmon [22], no significant differences between the proportion of motile spermatozoa were found between age groups. However, as mentioned above, those studies involved different fish per age group, while in the case of the present work the same breeding males were analyzed.

Evidently, age can be one of the influencing factors for sperm quality in farmed Arctic charr males. Temperature conditions during sperm development and spawning also play a crucial role [81, 83]. In the case of our study, the spawning temperatures (Fig. 2) were close during the two years of measurements though differences were observed. More specifically, the temperature in August (average: 13.8 °C, max: 14.9 °C) and September (average: 12.3°C, max: 13.4) 2022 was higher than in the previous year (August: 12.3°C, max: 13.4°C; September: 11.1°C, max:12.0°C; Supplementary Fig. S1). At the same time, the optimal temperature range for gamete development is considered to be below 12°C [3]. Therefore, the temperature fluctuations could have partially masked the true age effect of the recorded sperm quality parameters.

### Relationship between sperm quality traits and GEBVs

The direct comparison between 4- and 5-year-old males with 3-year-old ones was not possible due to different activation solutions used to induce sperm motility. Thus, the available GEBVs based on a single data measurement (October – November 2020) from the previous work [12] were used to provide a general understanding of relationships between estimated breeding values for sperm quality with the measured parameters in the subsequent spawning seasons (Table 3). In general, correlations between GEBVs and phenotypes have been widely used in animal selective breeding to assess the accuracy of the breeding values [84].

Notably, the correlation patterns between GEBVs and observed sperm concentration showed that the estimated genetic potential of the first-time-spawning males regarding sperm concentration had a moderate linear association with the recordings of the following two reproductive seasons. Concerning the motility traits, moderate linear associations with GEBVs were found only for velocities recorded in November. Aside from that, substantial differences were found between sampling points, suggesting that re-estimating GEBVs probably on a yearly basis might be required, at least for the motility-related traits. Another approach would be to combine data from several years, as in the case of livestock breeding, for higher milk production. Milk yield is a highly variable and heritable trait; thus, to ensure the most accurate selection of breeding candidates, the genomic evaluation for this trait utilizes average data across one or several years of lactation [85, 86].

### Underlying genomic regions associated with fluctuating sperm motility across the reproductive season

Considerable seasonal variability in fertility was suggested due to the substantial discrepancy of the recorded sperm quality traits among individual males and sampling points. The genome-wide *F*_*ST*_ detected genomic differentiation among males with high and low variability in total progressive motility across the spawning season.

In our previous study [12] using ddRAD-seq followed by GWAS for sperm quality traits, we highlighted a genomic region of interest on chromosome LG7. In the present study, we used WGS together with the a priori available ddRAD SNPs to scan for regions of genetic differentiation between animals of consistently high progressive motility versus ones with a variable one. Several overlapping regions (on chromosomes LG11, LG15, and LG18) were found between the two analyses. Moreover, candidate genes related to male performance during the reproductive season were suggested on four chromosomes (LG4q.1:29, LG11, LG14, and LG22). The gene ontology analysis pointed out several of those that could play a role in mammalian male fertility.

To the best of our knowledge, the genetic basis for sperm quality variability throughout the spawning season has not been studied in any fish species. Moreover, even though the genetic architecture of semen traits has been studied in livestock species using SNP genotyping [32] or whole-genome sequencing [36, 87], the possibility that sperm quality fluctuations across a reproductive season are at least partially controlled by genetic factors remains unknown. Notably, genetic effects for another dynamically fluctuating trait, as milk production in dairy suggested candidate genes with potential impact on the variation of milk production during or across lactations [88–90]. Furthermore, the above studies suggested that the effects of some of the quantitative trait loci (QTLs) were not constant within or across lactations.

Overall, our findings present novel insights regarding underlying genomic regions associated with fluctuations of sperm quality in farmed Arctic charr. At the same time, it should be admitted that the limited number of males used in our study may not be sufficient to provide a solid conclusion on identified SNPs and genomic regions of interest. Future studies with a larger sample size could shed additional light on the underlying genetic architecture.

## Conclusion

Arctic charr milt characteristics such as concentration and sperm motility parameters varied both within and amongst the spawning seasons. Individual performance differed, especially in terms of sperm motility throughout the reproductive seasons. Furthermore, the variability in sperm quality was evident on a family level. A reproductive pattern in terms of age suggested generally higher sperm motility and velocity records in 4-year-old males compared to the values obtained from the same animals one year later. GEBVs for sperm quality parameters had moderate and significant associations with sperm concentrations across the spawning season. Finally, genome-wide *F*_*ST*_ estimates provided the first insights into the underlying genomic factors associated with sperm quality fluctuation across the reproductive season.

### Electronic supplementary material

Below is the link to the electronic supplementary material.

Supplementary Material 1

## Data Availability

Sequenced reads in the form of fastq files were deposited to the National Centre for Biotechnology Information (NCBI) repository and are publicly available under the project ID PRJNA946925 (https://www.ncbi.nlm.nih.gov/bioproject/PRJNA946925).
