# Supplementary material for: Seasonal and age-related changes in sperm quality of farmed arctic charr (Salvelinus alpinus)
Source: BMC Genomics. 2023 Sep 4;24:519. doi: 10.1186/s12864-023-09614-9 (PMC10478403; doi:10.1186/s12864-023-09614-9)
Supplement: Supplementary file 1 — Supplementary Material 1 [file 12864_2023_9614_MOESM1_ESM.docx]

**Supplementary material**

**Table S1** Arctic charr (*Salvelinus alpinus*) family structure, number, and male ID with the natural and delayed spawning.

| Family no. | Dam ID | Sire ID | Male ID | |
| --- | --- | --- | --- | --- |
|  |  |  | Natural spawning | Delayed spawning |
| 1 | D1 | S9 | - | 44 |
| 2 | D2 | S37 | - | 33, 66 |
| 3 | D3 | S23 | 29, 33, 35, 36 | - |
| 4 | D4 | S21 | 25, 39 | 30, 51 |
| 5 | D5 | S7 | 7 | 14, 25 |
| 6 | D6 | S17 | 20, 27 | - |
| 7 | D7 | S36 | - | 27 |
| 8 | D8 | S33 | 59 | 59 |
| 9 | D9 | S29 | 42 | 17 |
| 10 | D10 | S25 | 32 | 26, 35, 37 |
| 11 | D11 | S39 | - | 64 |
| 12 | D12 | S2 | 28 | 32, 60 |
| 13 | D13 | S4 | - | 34 |
| 14 | D14 | S15 | 40, 49 | 19, 22, 39, 57 |
| 15 | D15 | S8 | 8, 9 | 5, 20, 65 |
| 16 | D16 | S27 | 43 | 15 |
| 17 | D17 | S12 | - | 16, 72 |
| 18 | D18 | S6 | 6 | - |
| 19 | D19 | S33 | 55 | 49 |
| 20 | D20 | S34 | 62 | 54 |
| 21 | D21 | S30 | 45 | 9, 28, 46 |
| 22 | D22 | S9 | 10 | - |
| 23 | D23 | S2 | 2 | 47 |
| 24 | D24 | S10 | 11 | 8, 29 |
| 25 | D25 | S35 | - | 23, 43, 62 |
| 26 | D26 | S27 | 38 | 18, 56 |
| 27 | D27 | S14 | 37 | 71, 73 |
| 28 | D28 | S1 | 1 | 3 |
| 29 | D29 | S19 | 23, 64 | - |
| 30 | D30 | S38 | - | 63, 68 |
| 31 | D31 | S5 | 5, 14 | - |
| 32 | D32 | S21 | 31, 48 | 58 |
| 33 | D33 | S12 | 13 | 1, 10, 11, 24, 40, 55, 61 |
| 34 | D34 | S32 | 50 | - |
| 35 | D35 | S11 | 12, 21, 51 | 4 |
| 36 | D36 | S31 | 46, 58 | 38 |
| 37 | D37 | S13 | 15 | 7, 48 |
| 38 | D38 | S26 | 34, 57 | 42 |
| 39 | D39 | S32 | 56, 63 | 70 |
| 40 | D40 | S18 | 22, 66 | - |
| 41 | D41 | S24 | 53 | 31, 69 |
| 42 | D42 | S20 | 24 | 36 |
| 43 | D43 | S15 | 18 | 53 |
| 44 | D44 | S19 | 65 | 52 |
| 45 | D45 | S3 | 3, 16 | - |
| 46 | D46 | S28 | 41 | 2 |
| 47 | D47 | S14 | 17 | 6, 13 |
| 48 | D48 | S8 | 47 | 12 |
| 49 | D49 | S22 | 26 | 41, 50 |
| 50 | D50 | S16 | 19, 54, 61 | 21 |
| 51 | D51 | S22 | - | 67 |
| 52 | D52 | S4 | 4, 44 | - |
| 53 | D53 | S24 | 30, 52, 60 | 45 |

**Table S2** CASA software settings for Arctic charr sperm analysis

| Parameter | Function | Settings |
| --- | --- | --- |
| Magnification | Microscope setting | 10x |
| Capture method | Microscope setting | Ph- |
| Grid distance (µm) | Microscope setting | 100 |
| Analysis timeout | Image capture | 20 |
| Box size (pixel) | Image capture | 152 |
| Frame rate (fps) | Image capture | 100 |
| Number of images | Image capture | 50 |
| Preview (seconds) | Image capture | 1 |
| Timer (seconds) | Image capture | 5 |
| Disposable chamber | Counting cells | CellVision |
| Chamber depth (µm) | Counting cells | 20 |
| Area min (µm^2^) | Cell detection | 1 |
| Area max (µm^2^) | Cell detection | 90 |
| Static VCL cut-off (µm/s) | Static cell detection | < 20 |
| Slow-Medium VCL cut-off (µm/s) | Progressive cell detection | 45 |
| Rapid VCL cut-off (µm/s) | Progressive cell detection | > 100 |
| Straightness cut-off (µm/s) | Progressive cell detection | 80 |
| VAP points (pixels) | Static cell detection | 5 |

**Table S3** Descriptive statistics for Arctic charr (*Salvelinus alpinus*) sperm quality parameters sampled multiple times across two consecutive spawning periods.

| Parameter | Natural spawning | | | Delayed spawning | | |
| --- | --- | --- | --- | --- | --- | --- |
|  | October early | October late | November | October late | November | December |
| **2021** | | | | | | |
| SC | 2.71±1.82 | 2.71±1.67 | 3.73±1.81 | 2.01±1.81 | 2.77±1.73 | 4.68±2.18 |
| PM | 76 ± 30 | 79 ± 24 | 84 ± 27 | 58 ± 40 | 73 ± 35 | 84 ± 22 |
| VCL | 129 ± 47 | 132 ± 36 | 144 ± 27 | 109 ± 59 | 134 ± 49 | 150 ± 34 |
| VAP | 97 ± 39 | 103 ± 32 | 111 ± 23 | 83 ± 52 | 102 ± 45 | 118 ± 29 |
| VSL | 77 ± 33 | 87 ± 39 | 89 ± 22 | 69 ± 46 | 82 ± 39 | 97 ± 26 |
| **2022** | | | | | | |
| SC | 1.27±1.72 | 2.72±1.88 | 5.05±2.61 | - | - | - |
| PM | 52 ± 39 | 76 ± 29 | 72 ± 24 | - | - | - |
| VCL | 99 ± 58 | 129 ± 30 | 125 ± 38 | - | - | - |
| VAP | 67 ± 52 | 95 ± 43 | 93 ± 33 | - | - | - |
| VSL | 48 ± 40 | 74 ± 35 | 73 ± 28 | - | - | - |

SC: sperm concentration, ×10^9^ cells/mL; PM: total progressive motility, %; VCL: curvilinear velocity, μm/s; VAP: average path velocity, μm/s; VSL: straight-line velocity, μm/s.

**Table S4** Parameter estimates and standard errors (in parentheses) of mixed-effects models for sperm quality metrics in Arctic charr (*Salvelinus alpinus*) males.

| **Model 1** | | | | | |
| --- | --- | --- | --- | --- | --- |
| Parameter | SC | PM | VCL | VSL | VAP |
| **Fixed effects** | | | | | |
| Intercept | 1.9*** (0.3) | 61.9*** (4.7) | 113.8*** (6.9) | 64.9*** (5.4) | 82.5*** (6.1) |
| Sampling in late October | 0.05 (0.2) | -0.8 (4.2) | -1.2 (6.0) | 7.5 (4.7) | 3.3 (5.3) |
| Sampling in November | 0.9*** (0.2) | 9.4* (4.2) | 18.6** (6.0) | 15.4** (4.7) | 17.6** (5.3) |
| Sampling in December | 2.7*** (0.3) | 21.9*** (5.5) | 35.3*** (7.8) | 31.5*** (6.14) | 35.1*** (6.9) |
| Photoperiod | 0.8** (0.3) | 14.4*** (3.9) | 15.1* (5.9) | 12.1** (4.5) | 14.2** (5.2) |
| **Random effects** | | | | | |
| Within-individual variability,σ^2^ (SD) | 1.8 (1.3) | 220.4 (14.9) | 542.8 (23.3) | 299.8 (17.31) | 417.3 (20.4) |
| Residual, (SD) | 1.4 (1.2) | 661.2 (25.7) | 1386.6 (37.2) | 840.1 (28.9) | 1072.5 (32.8) |
| ^1^**Model 2** | | | | | |
| Parameter | SC | PM | VCL | VSL | VAP |
| **Cross-level interaction** | | | | | |
| Intercept | 3.7*** (0.2) | 82.4*** (3.6) | 143.8*** (5.3) | 89.5*** (4.1) | 111.1*** (4.7) |
| Sampling in late October vs. Delayed photoperiod | -1.7*** (0.3) | -24.2*** (5.0) | -34.5*** (7.4) | -19.7*** (5.7) | -28.2*** (6.5) |
| Sampling in November vs. Delayed photoperiod | -0.9** (0.3) | -7.9 (5.0) | -7.9 (7.4) | -6.6 (5.7) | -8.0 (6.5) |
| Sampling in December vs. Delayed photoperiod | 0.9** (0.3) | 1.5 (5.1) | 5.4 (7.6) | 6.8 (5.9) | 6.5 (6.7) |
| Sampling in early October vs. Natural photoperiod | -1.0*** (0.2) | -6.0 (4.4) | -14.8* (6.5) | -12.6* (5.0) | -14.7* (5.7) |
| Sampling in late October vs. Natural photoperiod | -1.0*** (0.2) | -3.5 (4.4) | -12.2 (6.4) | -2.3 (5.0) | -7.9 (5.7) |
| **Random effects** | | | | | |
| Within-individual variability,σ^2^ (SD) | 1.8 (1.3) | 223.6 (14.9) | 546.9 (23.4) | 301.9 (17.3) | 420.1 (20.5) |
| Residual, (SD) | 1.4 (1.2) | 652.8 (25.6) | 1377.5 (37.1) | 835.3 (28.9) | 1066.3 (32.7) |
| **Model 3** | | | | | |
| Parameter | SC | PM | VCL | VSL | VAP |
| **Fixed effects** | | | | | |
| Intercept | -476.2 (358.2) | 24047.0*** (5566.7) | 31374.4*** (8161.3) | 38348.7*** (5909.0) | 35169.6*** (7046.9) |
| Sampling in late October | 0.6** (0.2) | 13.1*** (3.3) | 16.3*** (4.8) | 17.7*** (3.5) | 17.9*** (4.2) |
| Sampling in November | 1.9*** (0.2) | 12.8***(3.3) | 19.6*** (4.9) | 18.4*** (3.5) | 19.8*** (4.2) |
| Year | 0.2 (0.2) | -11.9*** (2.8) | -15.5*** (4.0) | -18.9*** (2.9) | -17.4*** (3.9) |
| **Random effects** | | | | | |
| Within-individual variability,σ^2^ (SD) | 1.8 (1.3) | 122.7 (11.1) | 419.5 (20.5) | 214.7 (14.7) | 336.2 (18.3) |
| Residual, (SD) | 2.5 (1.5) | 690.0 (26.3) | 1477.5 (38.4) | 775.4 (27.8) | 1097.8 (33.1) |

**P* < 0.05, ***P* < 0.01, ****P* < 0.001. Mixed effects **model 1** includes animal as random effect and sampling date and photoperiod as fixed effects. **Model 2** includes animal as random effect and interaction between sampling date and photoperiod as fixed effect. ^1^**Model 2** matrix was rank deficient. **Model 3** includes animal as random effect and year as fixed effect. **Model 1** and **model 2** was fitted for records of males from 2021 spawning year with natural and delayed spawning. **Model 3** was fitted for records of males from 2021 and 2022 spawning years with natural spawning.

**Table S5** Information criteria for mixed-effects model comparison used to estimate the parameter effects for sperm quality metrics in Arctic charr (*Salvelinus alpinus*) males.

| **Model 1** | | | | | |
| --- | --- | --- | --- | --- | --- |
| Parameter | SC | PM | VCL | VSL | VAP |
| AIC | 1545.5 | 3928.8 | 4241.7 | 4031.1 | 4136.7 |
| BIC | 1573.6 | 3956.9 | 4269.8 | 4059.2 | 4164.9 |
| **Model 2** | | | | | |
| AIC | 1547.4 | 3921.1 | 4234.9 | 4024.9 | 4130.3 |
| BIC | 1579.5 | 3953.3 | 4267.0 | 4057.1 | 4162.5 |
| **Model 3** | | | | | |
| AIC | 1509.9 | 3642.1 | 4012.7 | 3762.6 | 3901.6 |
| BIC | 1533.3 | 3665.8 | 4036.5 | 3786.4 | 3925.4 |

**Table S6** Mean total progressive motility (PM, %) and standard deviations (SD) measured across the spawning period in individual Arctic charr (*Salvelinus alpinus*) males with the natural and delayed spawning in two consecutive spawning seasons.

| Sampling 2021 | | | | | | | | Sampling 2022 | | | |
| --- | --- | --- | --- | --- | --- | --- | --- | --- | --- | --- | --- |
| Natural spawning | | | | Delayed spawning | | | | Natural spawning | | | |
| Male ID | Mean PM | SD | CV | Male ID | Mean PM | SD | CV | Male ID | Mean PM | SD | CV |
| 1 | 33 | 26 | 0,79 | 1 | 0 | 0 | - | 1 | 0 | 0 | - |
| 2 | 40 | 12 | 0,30 | 2 | 10 | 11 | 1,10 | 2 | 8 | 14 | 1,75 |
| 3 | 50 | 44 | 0,88 | 3 | 24 | 21 | 0,88 | 3 | 17 | 19 | 1,12 |
| 4 | 52 | 47 | 0,90 | 4 | 26 | 37 | 1,42 | 4 | 31 | 47 | 1,52 |
| 5 | 53 | 48 | 0,91 | 5 | 27 | 48 | 1,78 | 5 | 32 | 38 | 1,19 |
| 6 | 55 | 49 | 0,89 | 6 | 28 | 48 | 1,71 | 6 | 32 | 25 | 0,78 |
| 7 | 56 | 51 | 0,91 | 7 | 29 | 36 | 1,24 | 7 | 33 | 34 | 1,03 |
| 8 | 59 | 32 | 0,54 | 8 | 29 | 50 | 1,72 | 8 | 35 | 18 | 0,51 |
| 9 | 60 | 44 | 0,73 | 9 | 30 | 52 | 1,73 | 9 | 37 | 48 | 1,30 |
| 10 | 61 | 47 | 0,77 | 10 | 34 | 48 | 1,41 | 10 | 41 | 32 | 0,78 |
| 11 | 61 | 53 | 0,87 | 11 | 35 | 51 | 1,46 | 11 | 44 | 48 | 1,09 |
| 12 | 62 | 49 | 0,79 | 12 | 39 | 50 | 1,28 | 12 | 45 | 63 | 1,40 |
| 13 | 62 | 53 | 0,85 | 13 | 41 | 33 | 0,80 | 13 | 45 | 39 | 0,87 |
| 14 | 63 | 55 | 0,87 | 14 | 41 | 35 | 0,85 | 14 | 47 | 33 | 0,70 |
| 15 | 66 | 42 | 0,64 | 15 | 46 | 65 | 1,41 | 15 | 48 | 27 | 0,56 |
| 16 | 66 | 31 | 0,47 | 16 | 47 | 43 | 0,91 | 16 | 48 | 68 | 1,42 |
| 17 | 68 | 30 | 0,44 | 17 | 47 | 48 | 1,02 | 17 | 49 | 69 | 1,41 |
| 18 | 70 | 22 | 0,31 | 18 | 54 | 48 | 0,89 | 18 | 50 | 40 | 0,80 |
| 19 | 70 | 40 | 0,57 | 19 | 54 | 14 | 0,26 | 19 | 51 | 64 | 1,25 |
| 20 | 73 | 16 | 0,22 | 20 | 58 | 55 | 0,95 | 20 | 52 | 46 | 0,88 |
| 21 | 77 | 24 | 0,31 | 21 | 58 | 25 | 0,43 | 21 | 52 | 39 | 0,75 |
| 22 | 78 | 17 | 0,22 | 22 | 59 | 51 | 0,86 | 22 | 54 | 46 | 0,85 |
| 23 | 79 | 9 | 0,11 | 23 | 59 | 52 | 0,88 | 23 | 55 | 47 | 0,85 |
| 24 | 80 | 17 | 0,21 | 24 | 63 | 55 | 0,87 | 24 | 56 | 37 | 0,66 |
| 25 | 80 | 15 | 0,19 | 25 | 64 | 56 | 0,88 | 25 | 56 | 52 | 0,93 |
| 26 | 80 | 3 | 0,04 | 26 | 64 | 48 | 0,75 | 26 | 57 | 48 | 0,84 |
| 27 | 81 | 21 | 0,26 | 27 | 64 | 56 | 0,88 | 27 | 57 | 48 | 0,84 |
| 28 | 81 | 15 | 0,19 | 28 | 65 | 44 | 0,68 | 28 | 59 | 50 | 0,85 |
| 29 | 81 | 13 | 0,16 | 29 | 67 | 9 | 0,13 | 29 | 62 | 51 | 0,82 |
| 30 | 81 | 6 | 0,07 | 30 | 68 | 40 | 0,59 | 30 | 64 | 23 | 0,36 |
| 31 | 82 | 5 | 0,06 | 31 | 70 | 41 | 0,59 | 31 | 66 | 46 | 0,70 |
| 32 | 83 | 13 | 0,16 | 32 | 71 | 28 | 0,39 | 32 | 68 | 49 | 0,72 |
| 33 | 85 | 9 | 0,11 | 33 | 73 | 36 | 0,49 | 33 | 69 | 20 | 0,29 |
| 34 | 85 | 7 | 0,08 | 34 | 76 | 27 | 0,36 | 34 | 69 | 33 | 0,48 |
| 35 | 85 | 8 | 0,09 | 35 | 79 | 20 | 0,25 | 35 | 71 | 25 | 0,35 |
| 36 | 85 | 13 | 0,15 | 36 | 79 | 5 | 0,06 | 36 | 72 | 30 | 0,42 |
| 37 | 86 | 9 | 0,10 | 37 | 81 | 23 | 0,28 | 37 | 76 | 25 | 0,33 |
| 38 | 86 | 16 | 0,19 | 38 | 85 | 7 | 0,08 | 38 | 80 | 24 | 0,30 |
| 39 | 86 | 8 | 0,09 | 39 | 85 | 20 | 0,24 | 39 | 80 | 14 | 0,18 |
| 40 | 87 | 3 | 0,03 | 40 | 85 | 18 | 0,21 | 40 | 81 | 10 | 0,12 |
| 41 | 87 | 8 | 0,09 | 41 | 88 | 10 | 0,11 | 41 | 84 | 12 | 0,14 |
| 42 | 87 | 8 | 0,09 | 42 | 88 | 12 | 0,14 | 42 | 86 | 8 | 0,09 |
| 43 | 88 | 12 | 0,14 | 43 | 89 | 6 | 0,07 | 43 | 86 | 1 | 0,01 |
| 44 | 88 | 3 | 0,03 | 44 | 89 | 11 | 0,12 | 44 | 87 | 4 | 0,05 |
| 45 | 88 | 10 | 0,11 | 45 | 90 | 11 | 0,12 | 45 | 88 | 6 | 0,07 |
| 46 | 89 | 4 | 0,04 | 46 | 90 | 10 | 0,11 | 46 | 89 | 9 | 0,10 |
| 47 | 90 | 15 | 0,17 | 47 | 91 | 14 | 0,15 | 47 | 90 | 2 | 0,02 |
| 48 | 90 | 4 | 0,04 | 48 | 91 | 3 | 0,03 | 48 | 92 | 7 | 0,08 |
| 49 | 91 | 3 | 0,03 | 49 | 91 | 4 | 0,04 | 49 | 92 | 6 | 0,07 |
| 50 | 91 | 7 | 0,08 | 50 | 91 | 3 | 0,03 | 50 | 93 | 4 | 0,04 |
| 51 | 92 | 2 | 0,02 | 51 | 93 | 1 | 0,01 | 51 | 94 | 6 | 0,06 |
| 52 | 92 | 8 | 0,09 | 52 | 93 | 5 | 0,05 | 52 | 94 | 3 | 0,03 |
| 53 | 92 | 4 | 0,04 | 53 | 93 | 4 | 0,04 | 53 | 94 | 1 | 0,01 |
| 54 | 92 | 8 | 0,09 | 54 | 93 | 2 | 0,02 | 54 | 95 | 2 | 0,02 |
| 55 | 92 | 7 | 0,08 | 55 | 94 | 3 | 0,03 | 55 | 95 | 4 | 0,04 |
| 56 | 92 | 6 | 0,07 | 56 | 94 | 4 | 0,04 | 56 | 96 | 3 | 0,03 |
| 57 | 93 | 7 | 0,08 | 57 | 94 | 2 | 0,02 | 57 | 96 | 2 | 0,02 |
| 58 | 93 | 0 | 0,00 | 58 | 94 | 3 | 0,03 | 58 | 96 | 3 | 0,03 |
| 59 | 94 | 4 | 0,04 | 59 | 95 | 5 | 0,05 | 59 | 96 | 1 | 0,01 |
| 60 | 95 | 2 | 0,02 | 60 | 95 | 2 | 0,02 | 60 | 97 | 1 | 0,01 |
| 61 | 96 | 1 | 0,01 | 61 | 95 | 6 | 0,06 | 61 | 98 | 0 | 0,00 |
| 62 | 96 | 3 | 0,03 | 62 | 95 | 3 | 0,03 | - | - | - |  |
| 63 | 97 | 2 | 0,02 | 63 | 95 | 5 | 0,05 | - | - | - |  |
| 64 | 97 | 2 | 0,02 | 64 | 95 | 1 | 0,01 | - | - | - |  |
| 65 | 97 | 3 | 0,03 | 65 | 95 | 7 | 0,07 | - | - | - |  |
| 66 | 98 | 1 | 0,01 | 66 | 95 | 3 | 0,03 | - | - | - |  |
| - | - | - |  | 67 | 96 | 3 | 0,03 | - | - | - |  |
| - | - | - |  | 68 | 96 | 3 | 0,03 | - | - | - |  |
| - | - | - |  | 69 | 97 | 1 | 0,01 | - | - | - |  |
| - | - | - |  | 70 | 97 | 2 | 0,02 | - | - | - |  |
| - | - | - |  | 71 | 97 | 1 | 0,01 | - | - | - |  |
| - | - | - |  | 72 | 97 | 2 | 0,02 | - | - | - |  |
| - | - | - |  | 73 | 98 | 1 | 0,01 | - | - | - |  |


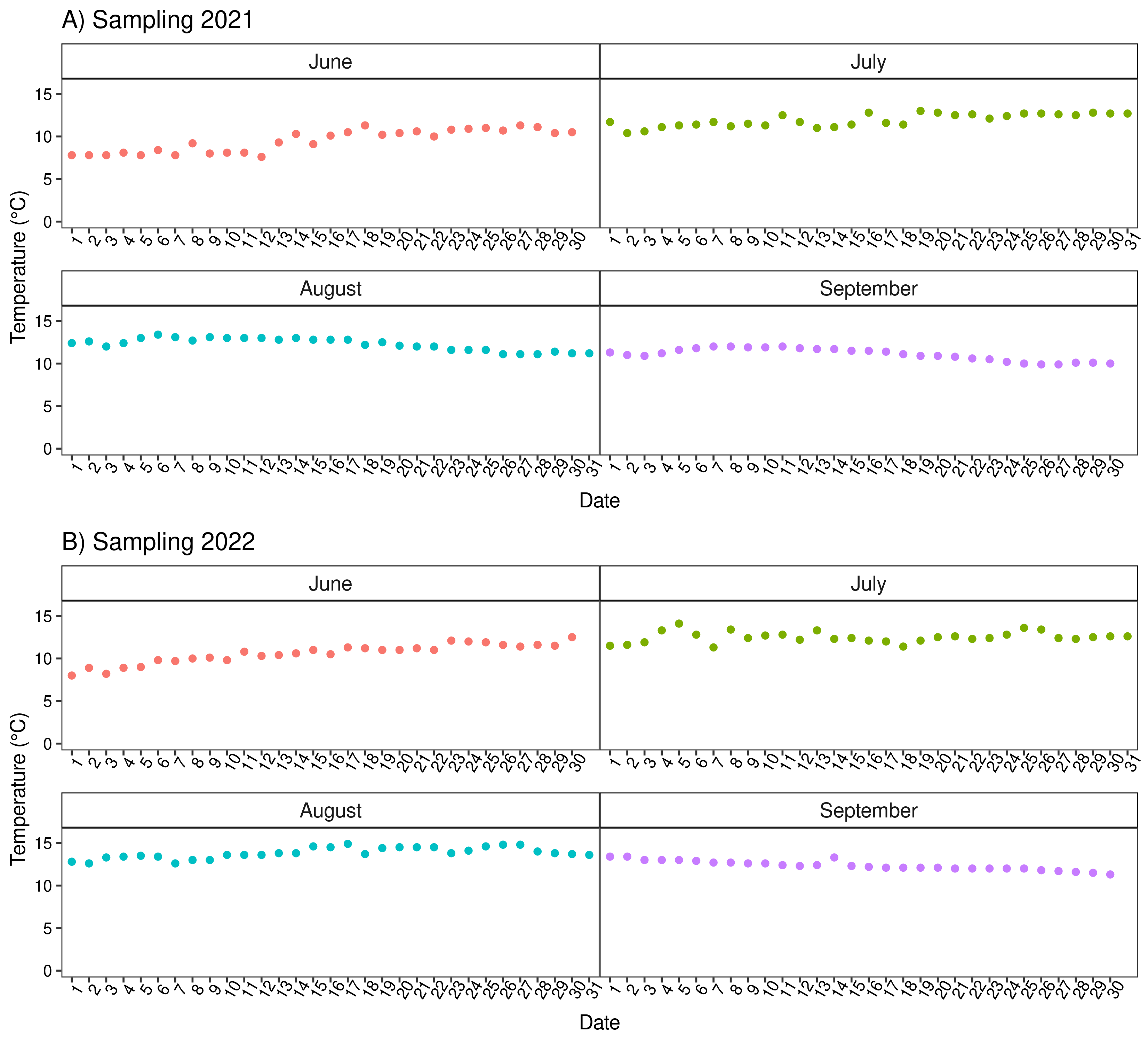


**Figure S1** Temperature regime during summer before the spawning season of farmed Arctic charr (*Salvelinus alpinus*) at sampling in 2021 (A) and 2022 (B).
